# Alcohol in Medicine

**Published:** 1854-06

**Authors:** 


### ALCOHOL IN MEDICINE.

We observe a sharp discussion has sprung up between our valued friend, Dr. Hall, of Carthage, Illinois, and a Dr. Fuller, in the Boston Medical Journal, on the subject of "Alcohol in Medicine." The disputants have waxed warm and sustain themselves well. Dr. Hall, we are pleased to see, wields his quill with gladiatorial skill, and shows himself a ready combatant. Some of his arguments, we will here venture the opinion, will be very difficult to disprove or overturn.—Dr. Hall is a young man of promise, and as he is devoted to his profession, will one day take a fore front rank in the profession. An original communication for this Journal from his pen, on "Professional Empiricism," has been extensively copied in many of the Journals, and in some of them highly commended. Press forward, young friend, for talent, worth, and merit, will ultimately triumph.

